# Problematic Exercise in Anorexia Nervosa: Testing Potential Risk Factors against Different Definitions

**DOI:** 10.1371/journal.pone.0143352

**Published:** 2015-11-30

**Authors:** Melissa Rizk, Christophe Lalanne, Sylvie Berthoz, Laurence Kern, EVHAN Group, Nathalie Godart

**Affiliations:** 1 CESP, INSERM, University Paris-Descartes, USPC, Paris, France; 2 University Paris Diderot, Paris Sorbonne Cité, EA 7334 (REMES), Patient-Centered Outcomes Research, Paris, France; 3 CESP, INSERM, University Paris-Sud, UVSQ, Université Paris-Saclay, Paris, France; 4 Psychiatry Unit, Institut Mutualiste Montsouris, Paris, France; 5 Laboratory EA 2931, CERSM, UFR-STAPS, Nanterre, France; Charité-Universitätsmedizin Berlin, Campus Benjamin Franklin, GERMANY

## Abstract

“Hyperactivity” has a wide prevalence range of 31% to 80% in the anorexia nervosa literature that could be partly due to the plethora of definitions provided by researchers in this field. The purpose of this study was two-fold: 1) To assess the variance across prevalence rates of problematic exercise encountered in patients with anorexia nervosa, in relation to seven different definitions found in the literature. 2) To examine how core eating disorder symptoms and the dimensions of emotional profile are associated with these different definitions and the impact of these definitions on the assessment of patients’ quality of life. Exercise was evaluated in terms of duration, intensity, type and compulsion using a semi-structured questionnaire administered to 180 women suffering from severe anorexia nervosa. Seven different definitions of problematic exercise were identified in the literature: three entailing a single dimension of problematic exercise (duration, compulsion or intensity) and four combining these different dimensions. Emotional profile scores, obsessive-compulsive symptoms, eating disorder symptomatology, worries and concerns about body shape, self-esteem and quality of life were assessed using several established questionnaires. The prevalence of problematic exercise varied considerably from, 5% to 54%, depending on the number of criteria used for its definition. The type and level of eating disorder symptomatology was found to be associated with several definitions of problematic exercise. Surprisingly, a better self-reported quality of life was found among problematic exercisers compared to non-problematic exercisers in three of the definitions. The different definitions of problematic exercise explain the broad prevalence ranges and the conflicting associations generally reported in the literature between problematic exercise and eating disorder-related psychological parameters. There is an urgent need for a valid consensus on the definition of problematic exercise in anorexia nervosa. This will support the development of further research on the etiology and treatment of problematic exercise.

## Introduction

Anorexia Nervosa (AN) is a severely debilitating eating disorder (ED) with considerable morbidity [1] and mortality [2]. With a chronicity incidence of 21% [3], it seems that, despite the best available treatment, many patients remain chronically ill [4]. Undoubtedly, the mechanisms triggering and maintaining this behavior are still insufficiently understood [5]. Patients suffering from ED have been found to have the highest frequency of physical activity and participation in sports compared to all other psychiatric disorders [6]. This “hyperactivity”, affecting 31% to 80% of AN patients [7], has been identified as a complex and multifaceted feature commonly present in AN. This wide range in prevalence range could be partly due to the plethora of definitions provided by researchers for patients with AN. Different terms, such as “hyperactivity” and “excessive exercise” have been used in the literature [8]. However, there is no international consensus on a clear and valid definition in AN. As the exercise involved is problematic, both in quantity (frequency, duration and intensity) and quality (compulsion to exercise), we will use the term “problematic exercise” (PE) in this paper and will carefully examine several ways of defining it. It should be noted that exercise is a subgroup of physical activity: it is a physical activity that is planned, structured, repetitive, and purposeful. General physical activity includes any body movement that contracts the muscles to burn more calories than the body would normally do at rest [9].

In a systematic review including 37 papers assessing physical activity in AN [10], PE was mainly evaluated using subjective methods centered on exercise (self-reports, semi-structured questionnaires, interviews, clinical charts or diaries (29 out of 37 studies)). It was less frequently evaluated using objective instruments that measure physical activity, and not solely exercise (14 out of 37 studies). Each of these methods has its own limitations. On the one hand, objective methods are based on complex methodologies and are therefore only used in small samples (on average 31 patients included in studies using accelerometers [11]). In addition, they can be difficult to implement, especially in an inpatient setting, leading to problems of refusal or compliance [11]. Subjective methods on the other hand, are mainly limited by the fact that they rely on patient recall. Patients, especially in AN, might give unreliable answers and/or deliberately omit to talk about PE as a symptom [12, 13]. Despite the fact they may underestimate physical activity [13–15], subjective methods seem easier to implement than an objective method in research that includes a large sample. Recently, Keyes et al. [16] found a significantly positive correlation between a subjective (international physical activity questionnaire) and an objective (actimetry) measure of physical activity in their AN sample.

Many studies have investigated factors that could explain PE in AN, with conflicting results. During the acute phase of AN, patients have been found to use physical activity as a coping strategy to compensate for, remove, and/or alleviate both negative affective states (anxiety [17, 18], depression [18] and stress [16, 19]) and ED symptoms [16] (including weight preoccupation [16, 20], drive for thinness |^21^–^23^], body dissatisfaction [21] and restrictive profile [24, 25]). Past research has mainly studied these elements separately or using different definitions of PE. Some authors have found links between PE and an early age at AN onset [26]. Quality of life also seems negatively impacted by the effect of PE interacting with ED severity [27]. In addition, alexithymia appears to have an important role in maintaining AN [28] by affecting the patient’s well-being [29]. To our knowledge, very few studies have simultaneously investigated all these factors and their implication in PE in AN. Furthermore, no study has taken into account the various qualitative characteristics (compulsive exercise) and/or the quantitative characteristics of exercise (frequency, duration and intensity) to investigate their association with the different predictive factors identified.

The purpose of this study was two-fold: 1) to assess the variance of prevalence rates of PE based on seven different definitions found in the literature. 2) To examine how ED symptoms, emotional profile and quality of life scores are associated with PE according to these different definitions.

## Methods

### Procedure and Ethics

This study was part of a larger longitudinal multi-centered study named EVHAN (Evaluation of Hospitalization for AN, Eudract number: 2007-A01110-53, registered in Clinical trials). The study protocol was approved by the Ile-de-France III Ethics Committee and the CNIL (Commission nationale de l’informatique et des libertés). In accordance with the Helsinki declaration, written informed consent was obtained from each patient before inclusion, and from the parents of those who were under 18 years old. Both patients (either adults or children and their parents) gave a written consent.

Prior to inclusion in the study, all participants were hospitalized in an inpatient care unit for life-threatening physical and/or mental states (including a body mass index (BMI) below 14 and/or rapid weight loss and/or compromised vital functions, severe depression, high suicide risk, chronic under-nutrition with low weight, and/or failure of out-patient care). The EVHAN inclusion criteria were patients aged 8 to 65 years old referred for an acute anorexia nervosa episode to one of the 11 French specialized inpatient treatment facilities participating in the EVHAN study. Individuals were excluded if they: 1) refused to participate in the research; 2) had insufficient knowledge of the French language; 3) were suffering, in addition to their ED, from potentially confounding somatic pathologies (diabetes, Crohn’s disease, or metabolic disorders); 4) had already been included in the protocol during a previous hospitalization.

### Participants

A total sample of 233 patients was included in the EVHAN study between April 2009 and July 2012. Current AN diagnosis was based on the DSM-IV-TR criteria (APA, 2000) and assessed using the CIDI 3.0 [30]. The following BMI criteria were applied: BMI < 10th percentile up to 17 years of age, and BMI < 17.5 for 17 years of age and older. Purging symptoms were evaluated using the Eating Disorder Examination Questionnaire (EDE-Q v. 5.2) [31]. At inclusion: (1) seven patients did not meet DSM-IV-TR criterion A. However, two of them had shifted from a BMI above the 97th percentile to a BMI on the 10th percentile relative to their age in the three months preceding hospitalization. The remaining five had had a BMI < 17.5 in the previous three months but had been initially admitted to a medical ward. They had gained weight just before their transfer to a psychiatry unit and inclusion in the study; (2) 39 patients did not meet DSM-IV-TR criterion B; (3) 16 patients did not meet DSM-IV-TR criterion C; (4) 10 patients did not meet DSM-IV-TR criterion D. We considered all the patients (AN full syndrome and sub-threshold) in our analyses.

The exclusion criteria for the present analyses were male gender (n = 10) and age younger than 13 years (n = 11) (age from which all patients had the same versions of the questionnaires of interest). Participants who had significant missing data for the variables of interest (n = 32) were also excluded.

### Measures

All data were collected during the first 2 weeks of inpatient admission.

#### Anthropometry

Body weight and height were measured using standard beam balance scales (Omega-SECA, Germany) and a stadiometer (wall mounted model 222-SECA, Germany) respectively. BMI was derived from weight (kg) divided by the square of height (meters) [32].

#### Emotional profile

Anxiety and depressive symptoms were evaluated using the Hospital Anxiety and Depression scale (HADS) [33]. In the present analyses, a summed scale score for anxiety and depression (ranging from 0 to 42 points) was calculated. The higher this composite score, the higher the level of anxiety and depressive symptoms reported by the subject.

Difficulties in emotional introspection and regulation were measured using the Bermond—Vorst Alexithymia Questionnaire-Form B (BVAQ-B) [34]. This is a 20-item questionnaire that includes five subscales: verbalizing emotional experiences (B1), daydreaming and fantasies (B2), identifying emotions (B3), proneness to being aroused by emotion inducing events (B4), and analyzing one’s own emotional states and reactions (B5). We used the BVAQ-B total score; a higher score indicates a higher level of alexithymia.

Self-esteem was measured using the 10-item version of the Rosenberg Self-Esteem Scale (RSES) [35]. A higher score indicates better self-esteem.

#### Obsessive-compulsive symptoms

Obsessive-compulsive symptoms were assessed using the Maudsley Obsessive Compulsive Inventory (MOCI) [36]. It includes 30 items and four subscales: Checking compulsions, Washing/cleaning compulsions, Slowness, and Doubting. The MOCI total score was used in the present analyses. A higher score indicates higher levels of obsessive-compulsive symptoms.

#### Body shape concerns

The body shape questionnaire (BSQ) [37] is a 34-item questionnaire used to evaluate worries and concerns about body shape. The BSQ total score was used in the present analyses. A higher score indicates marked worries and concerns about body shape.

#### Eating disorder symptomatology

ED symptoms were assessed using two subscales from the 26-item Eating Attitudes Test (EAT-26): the dieting and bulimia and the food preoccupation subscales [38]. The present analyses included these two subscales because they were the only ones associated with PE in the literature [24, 25].

#### Quality of life

Quality of life was evaluated with the Eating Disorders Quality of Life (EDQOL) scale. It includes 25 items and four subscales: Psychological, Physical/Cognitive, Work/School and Financial [39]. A higher score indicates poorer quality of life.

#### Exercise: duration, intensity and type

Participants were interviewed by trained evaluators, using a semi-structured questionnaire. This questionnaire was designed to ascertain at what level patients were engaging in a given type of exercise in the month preceding hospitalization. It was intended to evaluate the type of exercise (walking, running, swimming, cycling and household activities), frequency and duration (in hours per week). At the end of the questionnaire, in an open question, patients were asked to specify any other activity they were practicing. Each exercise pattern was then matched with its intensity in metabolic equivalents (METs) using the compendium of physical activity proposed by Ainsworth et al. [40]. The MET value of each physical activity represents the ratio of the energy expended per kilogram of body weight per hour during the activity compared to the energy expended when sitting quietly. The number of hours spent per day on each activity was multiplied by its MET score. The daily amount of exercise was then obtained by summing the MET-hours for all activities. Mean intensities of exercise were categorized under light-intensity (1.1 to 2.9 METs), moderate-intensity (3.0 to 5.9 METs) and vigorous-intensity (6.0 to 10 METs) (appendix 1 [41]). These cut-offs have been frequently used in other studies [14, 42–44]. Exercises were also classified into two types: individual and team sports [45]. Individual sports included walking, jogging, cycling, swimming, long-distance running, body-building, esthetics-oriented sports, martial arts, and technical or adrenaline sports.

#### Problematic exercise

**Fig 1:**
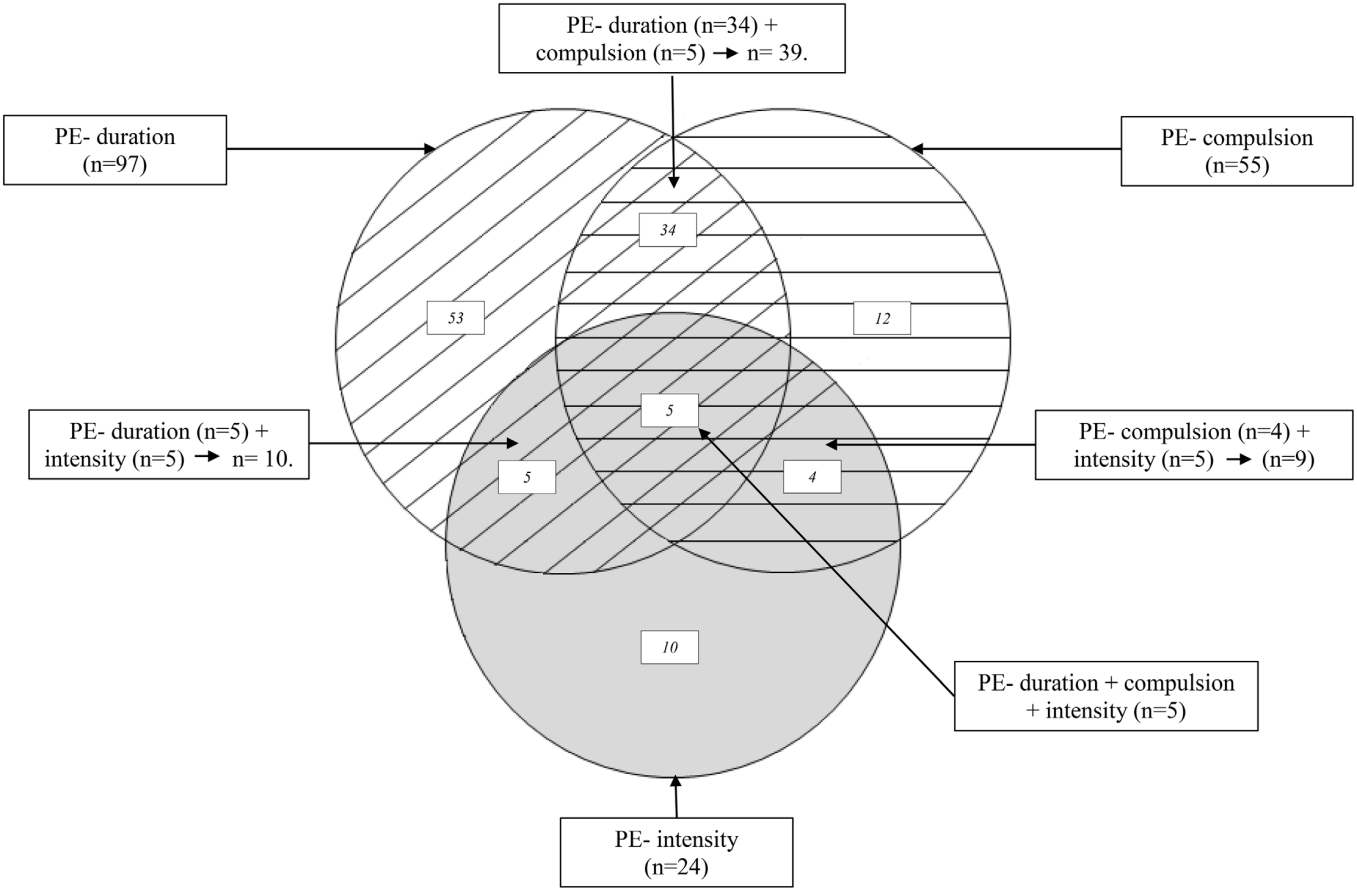
Seven problematic exercise (PE) definitions and the number of problematic exercisers included in each definition and subgroup. (n) Number of patients included in each subgroup.

We identified seven definitions of PE based on the quantitative (duration and intensity of physical activity) or qualitative (compulsion to exercise) dimensions implemented by studies in the literature and the instruments they used to evaluate these dimensions [25, 46–59] (S1 Table). The first three definitions were based on a single quantitative or qualitative criterion. The fourth, fifth and sixth definitions included combinations of two of the first three definitions. The seventh definition combined all three (Fig 1):

- The first definition, labeled “PE-duration”, was based on the duration of exercise per week. In this case, a patient was considered a problematic exerciser if she exercised for at least six hours a week before admission. This duration criterion is the most commonly used in the literature to assess excessive exercise in AN (S1 Table).
- The second definition, labeled “PE-compulsion”, was based on compulsion to exercise. In this case, the frequency of compulsion to exercise for reasons of weight or shape was assessed from item 18 in the EDE-Q [60]. Specifically, episodes of compulsion to exercise were calculated on the basis of the participants’ answer to the question: “over the past 28 days, how many times have you exercised in a restraining or compulsive way in order to control your weight or the shape of your body, to eliminate fat or to burn calories?” [61]. In this case, patients were considered problematic exercisers if they exercised for reasons of weight or shape more than five times a week in the 28 days prior to assessment [61–63].
- The third definition, labeled “PE-intensity”, was based on the intensity of exercise. In this case, a patient was considered a problematic exerciser if she exercised at a vigorous intensity level (≥ 6.0 METs).
- The fourth definition, labeled “PE-duration+compulsion”, was a combination of a quantitative criterion (duration) and a qualitative criterion (compulsion): in this case, patients were considered problematic exercisers if they exercised for more than 6 hours a week *and* exercised for reasons of weight or shape more than five times a week in the 28 days prior to assessment.
- The fifth definition, labeled “PE-duration+intensity”, was a combination of two quantitative criteria: here, patients were considered problematic exercisers if they exercised for more than 6 hours a week *and* at a vigorous intensity level.
- The sixth definition, labeled “PE-compulsion+intensity”, was a combination of a quantitative criterion (intensity) and a qualitative criterion (compulsion): in this case, patients were considered problematic exercisers if they exercised at a vigorous intensity level *and* exercised for reasons of weight or shape more than five times a week in the 28 days prior to assessment.
- The seventh definition, labeled “PE-duration+compulsion+intensity”, was a combination of two quantitative criteria (duration and intensity) and one qualitative criterion (compulsion): in this case, patients were considered problematic exercisers if they exercised for more than 6 hours a week *and* at a vigorous intensity level *and* exercised for weight or shape reasons more than five times a week in the 28 days prior to assessment.

### Statistics

A statistical analysis was performed using SPSS software (SPSS Statistics, version 21.0; Chicago) and R 3.1.2 (R statistical programing language version 3.2.1). Numerical variables were summarized as mean and standard deviation, while counts and frequencies were used for categorical variables. Group differences were tested using the appropriate univariate analyses (the Student t-test for independent samples, the Wilcoxon signed rank test for paired samples and the Welch t-test for continuous outcomes). A fixed Type I error of 5% was considered.

A series of multivariate logistic regressions was used to further explain the seven definitions of PE (mentioned above) in relation to our potential explanatory variables of interest. The following explanatory variables were considered in each model: Age, BMI, Illness duration, AN subtype, EAT dieting and bulimia subscale scores (ED symptomatology), BSQ score (body image), MOCI total score (obsession and compulsion), HADS Anxiety and Depressive symptoms summed as a single score, Rosenberg score (self-esteem), and BVAQ-B total score (alexithymia).

The seventh definition was excluded from our statistical analysis on account of the very small number of patients involved (n = 5). The outcomes considered in the remaining six multivariate models were respectively: 1) PE-duration for model 1; 2) PE-compulsion for model 2, 3) PE-intensity for model 3; 4) PE duration + compulsion for model 4, which is a combination of models 1 and 2; 5) PE duration + intensity for model 5, which is a combination of models 1 and 3. 6) PE compulsion + intensity for model 6, which is a combination of models 2 and 3. Because of the large number of predictors in relation to the number of positive cases in each model, two approaches were undertaken to select relevant markers in the set of predictors. First, a Lasso (L1 norm or Lasso) penalization was applied to the parameters of the logistic regression models in order to provide sparse solutions by zeroing low-contributing variables. A tuning of model hyper-parameters (penalty) was performed using repeated 10-fold cross-validations (25 repetitions). The final solution chosen was the one that most minimized the cross-validated log-likelihood on the optimal penalty parameter using a hybrid algorithm. It combined Newton’s method and a gradient descent, implemented in the penalized R package [64]. To assess the stability of this Lasso solution, bootstrap resampling (1000 samples) was used to estimate the 2.5 and 97.5 quantiles of the empirical distribution of regression coefficients for all selected variables. These solutions were then compared with those obtained from a simple stepwise backward model selection using the AIC criterion. This was enhanced by a bootstrap procedure (200 samples) to assess the variability of the final set of selected covariates. Finally, the number of times each covariate was retained in the final model and the frequency with which their regression coefficient was found statistically significant at a 5% level were computed on all bootstrap models.

## Results

### Participants’ characteristics

Our final sample consisted of 180 inpatients. There were no statistically significant differences between excluded patients and patients retained for further analyses for age (p = 0.745), BMI (p = 0.817), illness duration (p = 0.296), AN subtype (p = 0.314), EDE-Q scores (item 18, p = 0.206), total hours of exercise per week (p = 0.982), and HADS anxiety (p = 0.236) or depression (p = 0.211).

At admission to inpatient treatment, the mean age of patients was 20.7 years (SD = 5.9), their mean BMI was 14.3 (SD = 1.5) and the mean illness duration was 4.3 years (SD = 4.4). Regarding AN subtypes, 85 (47.2%) were restrictive AN (AN-R) and 95 (52.8%) were binge-eating/purging AN (AN-BP). Characteristics at inclusion and global scores on psychological scales are presented in Table 1. Compared to AN-BP patients, AN-R patients had a lower BMI (13.9±1.3 vs 14.7± 1.6, p<0.001), lower scores of EAT-26 dieting (17.6±10.9 vs 22.0±9.9, p<0.1), EAT- 26 bulimia (6.0±3.4 vs 8.7±4.6, p<0.001) and BSQ (107.1±37.2 vs 123.3±37.2, p<0.01).

**Table 1:** Patient characteristics (n = 180) at inclusion and global scores on psychological scales.

	Mean	SD	Minimum	Maximum
**BMI (kg/m** ^**2**^ **)**	14.3	1.5	10.3	18.9
**Age (years)**	20.7	5.9	13.4	43.8
**Illness duration (years)**	4.3	4.4	.22	24.4
**MOCI**	11.9	5.3	2.0	26.0
**BSQ**	115.8	37.9	34.0	194.0
**RSES**	11.0	5.0	3.0	24.0
**EAT-26 dieting**	19.9	10.5	0	39.0
**EAT-26 bulimia**	7.4	4.2	0	17.0
**HAD anx.dep**	22.1	7.4	17.0	27.5
**BVAQ-B**	56.4	9.0	34.0	80.0
**EDE-Q compulsive**	16.1	41.8	0	497
**Duration PA (hours/week)**	9.0	10.2	0	60.0
**Intensity PA (METs)**	3.6	2.3	0	10.0

BMI: Body mass index. BSQ: Body Shape Questionnaire score. BVAQ-B: Bermond—Vorst alexithymia questionnaire-form B. Duration PA: Total duration of physical activity. EAT-26 bulimia: 26-item Eating Attitudes Test bulimia and food preoccupation subscale score. EAT-26 dieting: 26-item Eating Attitudes Test dieting subscale score. EDE-Q compulsive: Item 18 of the EDE-Q. HAD anx.dep: Hospital anxiety and depression scale composite score. Intensity PA: Mean intensity of physical activities. MOCI: Maudsley obsessive-compulsive inventory total score. RSES: Rosenberg self-esteem scale score.

### Type of exercise

Walking was the most frequently reported exercise for both AN-R (72.9%) and AN-BP (66.3%). For AN-R, it was followed by cycling (27%), swimming (18.8%) and running (16.5%). For AN-BP, walking was followed by running (26.3%), cycling (22.1%) and swimming (17.9%). Patients mostly preferred individual sports (97.9%) to team sports (2.1%).

### Prevalence of PE

Fifty-four percent (n = 97), 30.4% (n = 55), 13.3% (n = 24), 21.7% (n = 39), 5.5% (n = 10) and 5% (n = 9) of participants were classified as problematic exercisers in model 1, 2, 3, 4, 5 and 6 respectively (Fig 1).

### Associations between definitions of PE and ED symptoms, emotional profile and quality of life

#### Univariate analyses

When PE was defined using PE-duration, PE-compulsion and PE-duration+compulsion, compared to non-problematic exercisers, problematic exercisers had significantly higher MOCI, BSQ and EAT-26 dieting scores. They also had significantly higher EAT-26 bulimia scores when PE was defined using PE-compulsion (Table 2). When PE was defined using PE-duration and PE-duration+compulsion, problematic exercisers had significantly lower RSES scores than non-problematic exercisers. No between-group differences were observed for HADS and BVAQ-B scores for any of the PE definitions.

**Table 2:** Associations between problematic and non-problematic exercisers and ED symptoms, emotional profile and quality of life according to definition of problematic exercise.

	PE-duration	PE-compulsion	PE- intensity	PE-duration+ compulsion	PE-duration+ intensity	PE-compulsion+ intensity
**MOCI**	t = 3.2*	t = 3.3**	NS	t = 3.0*	NS	NS
**BSQ**	t = 3.6^†^	t = 4.3^†^	NS	t = 3.5**	NS	NS
**RSES**	t = -2.1*	NS	NS	t = -2.0*	NS	NS
**EAT-26 dieting**	t = 3.9^†^	t = 5.4^†^	NS	t = 4.4^†^	NS	NS
**EAT-26 bulimia**	NS	t = 2.5*	NS	NS	NS	NS
**HAD anx.dep**	NS	NS	NS	NS	NS	NS
**BVAQ-B**	NS	NS	NS	NS	NS	NS
**EDQOL subscales:**						
**Psychological**	t = -3.0*	t = -3.5**	NS	t = -2.4*	NS	NS
**Physical/cognitive**	t = -2.2*	t = -2.7*	NS	NS	NS	NS
**Financial**	t = -3.2*	t = -2.7*	NS	t = -2.9*	NS	NS
**Work/School**	NS	NS	NS	NS	NS	NS
**Total score**	t = -3.3**	t = -3.4**	NS	t = -2.6*	NS	NS

t: t values determined using the t-test for independent samples.

* p ≤ 0.05;

**p ≤ 0.01;

^†^≤ 0.001.

BSQ: Body Shape Questionnaire score. BVAQ-B: Bermond—Vorst alexithymia questionnaire-form B. EAT-26 bulimia: 26-item Eating Attitudes Test bulimia and food preoccupation subscale score. EAT-26 dieting: 26-item Eating Attitudes Test dieting subscale score. EDQOL: Eating Disorders Quality of Life score. HAD anx.dep: Hospital anxiety and depression scale composite score. MOCI: Maudsley obsessive-compulsive inventory total score. NS: Not significant. RSES: Rosenberg self-esteem scale score.

Concerning quality of life, problematic exercisers defined by PE-duration, PE-compulsion and PE-duration+compulsion had significantly lower EDQOL total scores than non-problematic exercisers (Table 2). Problematic exercisers had significantly lower scores on the psychological and financial subscales compared to non-problematic exercisers when PE was defined by PE-duration, PE-compulsion and PE-duration+compulsion. They also had significantly lower scores on the physical/cognitive subscale when PE was defined by PE-duration and PE-compulsion.

#### Multivariate analyses

The results of the series of multivariate regressions are presented in Table 3. This table only includes significant variables. Age and BMI were retained in all models as adjustment variables.

**Table 3:** Series of multivariate regressions for each definition of problematic exercise (PE): significant predictive factors^1^.

	PE-duration (n = 97)			PE-compulsion (n = 55)			PE-intensity (n = 24)			PE-duration + compulsion (n = 39)			PE-duration + intensity (n = 10)			PE-compulsion + intensity (n = 9)		
	Unp. c. (SE) [% selection]	OR [95% CI]	Pen. c. [95% range]	Unp. c. (SE) [% selection]	OR [95% CI]	Pen. c. [95% range]	Unp. c. (SE) [% selection]	OR [95% CI]	Pen. c. [95% range]	Unp. c. (SE) [% selection]	OR [95% CI]	Pen. c. [95% range]	Unp. c. (SE) [% selection]	OR [95% CI]	Pen. c. [95% range]	Unp. c. (SE) [% selection]	OR [95% CI]	Pen. c. [95% range]
**Intercept**	-3.37 (1.72) [100]			-4.73 (1.88) [100]			-0.14 (2.45) [100]			-6.62 (2.13) [100]			-6.40 (3.55) [100]			-1.28 (3.89) [100]		
**Age**	0.06* (0.03) [100]	1.06 [1.00; 1.13]	0.03 [-0.01; 0.10]	0.02 (0.03) [100]	1.02 [0.95; 1.08]	-0.02 [-0.07; 0.06]	-0.09^†^ (0.05) [100]	0.91 [0.82; 0.10]	0.10 [-0.28; 0.01]	0.04 (0.03) [100]	1.04 [0.97; 1.11]	-0.01 [-0.06; 0.08]	-0.06 (0.07) [100]	0.94 [0.81; 1.06]	-0.07 [-0.29; 0.11]	-0.05 (0.08) [100]	0.95 [0.80; 1.08]	-0.06 [-0.38; 0.10]
**BMI**	0.06 (0.11) [100]	1.06 [0.86; 1.32]	-0.08 [-0.15; 0.24]	0.11 (0.12) [100]	1.11 [0.89; 1.40]	-0.10 [-0.14; 0.34]	0.12 (0.16) [100]	1.13 [0.83; 1.53]	0.0 [-0.22; 0.36]	0.16 (0.13) [100]	1.17 [0.91; 1.52]	-0.14 [-0.11; 0.38]	0.21 (0.21) [100]	1.23 [0.81; 1.84]	-0.10 [-0.19; 0.57]	0.00 (0.25) [100]	1.00 [0.61; 1.61]	-0.12 [-0.80; 0.52]
**EAT diet.**	0.08** (0.02) [80.5]	1.09 [1.04; 1.14]	0.03 [0.00; 0.06]	0.10** (0.03) [89]	1.11 [1.05; 1.17]	0.05 [0.01; 0.08]	0.07^†^ (0.04) [61]	1.07 [1.00; 1.15]		0.11** (0.03) [92.5]	1.12 [1.06; 1.19]	0.04 [0.01; 0.08]				0.11* (0.05) [81.5]	1.12 [1.01; 1.25]	
**EAT bulim.**	-0.14* (0.06) [73]	0.87 [0.78; 0.97]		-0.12* (0.06) [76.5]	0.89 [0.78; 1.00]					-0.16* (0.07) [77]	0.85 [0.74; 0.97]							
**HAD anx.dep**													0.07 (0.05) [78.5]	1.08 [0.98; 1.20]				
**MOCI**	0.07* (0.03) [73]	1.07 [1.00; 1.14]	0.02 [0.00; 0.09]	0.07^†^ (0.04) [65]	1.07 [0.99; 1.15]	0.01 [0.00; 0.09]	-0.07 (0.05) [58]	0.93 [0.84; 1.02]		0.08 (0.04) [72]	1.08 [1.00; 1.17]	0.01 [0.00; 0.10]						
**BSQ**							-0.02^†^ (0.01) [65.5]	0.98 [0.96; 1.00]								-0.03 (0.01) [70.5]	0.97 [0.95; 1.01]	
**AIC**	235.5			203.8			143.0			170.6			81.9			75.97		
**LL**	-111.7		-117.7	-95.9		-102.1	-65.5		-68.7	-79.3		-88.0	-36.9		-39.1	-33.0		-35.5

^†^ p < .10,

* p < .05,

** p < .001.

^1^ Illness duration, anorexia nervosa subtypes, self-esteem and alexithymia were not significant in any of our six models.

AIC: Akaike Information Criterion; BMI: Body Mass Index; BSQ: Body Shape Questionnaire score; CI: Confidence Interval; EAT bulim.: 26-item Eating Attitudes Test bulimia and food preoccupation subscale scores. EAT diet.: 26-item Eating Attitudes Test dieting subscale score. HAD anx.dep: Hospital anxiety and depression scale composite score; LL: log-likelihood; MOCI: Maudsley obsessive-compulsive inventory total score; OR: Odds ratio; Pen. c.: Penalized coefficient; SE: Standard error; Unp. c.: Unpenalized coefficient.

In model 1 (PE-duration), problematic exercisers were significantly older, and had higher EAT-dieting scores and MOCI total scores than non-problematic exercisers. However, they had lower EAT-bulimia scores. In models 2 (PE-compulsion) and 4 (PE-duration+compulsion), problematic exercisers also had significantly higher EAT-dieting scores and lower EAT-bulimia scores than non-problematic exercisers. This difference in EAT-dieting scores was also found in model 6 (PE-compulsion+intensity). There was a trend for problematic exercisers to have higher MOCI total scores in model 2 (PE-compulsion). In model 3 (PE-intensity), besides a tendency for higher EAT-dieting scores, problematic exercisers were younger and had lower BSQ total scores compared to non-problematic exercisers.

No significant differences were found between problematic and non-problematic exercisers concerning illness duration, AN subtype, RSES, BVAQ-B, HADS and EDQOL scores in any of the models studied.

## Discussion

On the basis of a literature review of all the definitions previously given for PE in AN, our study was conducted for the following purposes: 1) to determine the prevalence of PE in AN according to seven different definitions: three definitions implementing a single dimension of PE (duration, compulsion or intensity) and four definitions combining these dimensions (three combining two and one combining the three dimensions); 2) to study simultaneously, across six different PE definitions, the impact of different factors that have been linked to PE in the literature, something that has never been done before.

During the acute phase of AN, almost all our patients indulged in an individual sport rather than a team sport (97.9% vs 2.1% respectively). Adolescents with ED have previously been found to participate more in individual sports than adolescents suffering from any other psychiatric disorder (even after adjusting for gender, age and socioeconomic status) [6]. In addition, social avoidance seems to be common in AN, and difficulties in social adaptation have been observed particularly in leisure activities [28]. Patients suffering from AN tend to avoid other people’s gaze and feel they are constantly judged on their physical appearance. In addition, taking part in team sports (i.e. football and basket) is highly dependent on others, which is not the case for individual sports. Walking was the most popular sport in our sample. This concurs with the few studies that assessed the types of exercise in their AN samples [16, 65]. Walking seems to be an accessible and practical way to exercise even when patients are severely emaciated and/or hospitalized (despite the direct and/or indirect restrictions of exercising imposed by a hospital environment [66]).

One of our main results was that the prevalence of PE varied considerably, as expected, from 5% to 54%, according to the definition used. This variation therefore results from on the criteria used to define PE. The most stringent definition, combining two quantitative criteria (duration and intensity) and one qualitative criterion (compulsion), identified the smallest proportion of problematic exercisers (n = 5). Conversely, the least stringent definition, entailing only a duration cut-off, identified the largest number of problematic exercisers (n = 97). This approach clearly highlights a considerable overlap of patients assigned to two or more definitions of PE (Fig 1). We notice, for example, that PE-duration and PE-compulsion have a huge overlap of participants of 40.2% (n = 39/97) and 70.9% (n = 39/55) respectively. Thus, including one or more criteria in PE definitions has major consequences on prevalence results. This could considerably affect the conclusions of studies assessing PE but using different definitions. To our knowledge, no one has previously studied the impact of different PE definitions on the same study sample. This is a very important result, since it could contribute to understanding why previous studies on PE in AN are not comparable: the problematic exercisers included in the different samples were partially or completely different. Without a valid common definition of PE, each author subjectively defined PE in the way he/she thought was appropriate for his/her study sample.

Interestingly, we found that patients exercising for more than 6 hours a week (PE-duration) generally scored higher on the compulsion item of the EDE-Q (12.0±10.5) compared to other patients (6.3±9.1). To evaluate the duration of a 6-hour a week physical activity as an efficient cut-off value for PE in AN, a Spearman rank correlation between scores for this item and the total number of hours of physical activity was estimated at 0.324 (p < 0.001). Then, a ROC analysis suggested that this cut-off value (the most widely used in the literature following the work of Davis and Fox [67]) seemed the best compromise in terms of sensitivity (0.672) and specificity (0.597) when one considers compulsion to exercise status derived from response to item 18 on the EDE-Q. The area under the curve (0.674) was comparable after adjusting for illness duration, BMI, and AN subtype (0.687).

When all potentially linked factors described in the literature were considered simultaneously, the various definitions of PE were significantly associated with different combinations of factors. The type and level of ED symptomatology (high restriction and/or low bulimia) were associated with the majority of our six models, except for model 5 (PE-duration+intensity) where the sample was very small. It is worth noting that AN subtypes were not linked to any PE definition. Thus, problematic exercisers tend to have considerable food restrictions and/or little bulimic behavior, regardless of PE definition and AN subtype. This result is in accordance with the study by Holtkamp et al. [17] study who found that food restriction was positively related to higher levels of exercise in their AN sample, significantly contributing to its variance (along with anxiety). Furthermore, Brewerton et al. [68] found that problematic exercisers suffering from AN or from bulimia nervosa (and defining PE as “exercise to control weight at least once a day and for at least 60 minutes”) were significantly less likely to show bulimic behaviors, such as binge eating, vomiting or using laxatives, than non-problematic exercisers. One hypothesis is that PE could be a consequence of food restriction. In findings on animal models, a reduction in food intake was also followed by a progressive increase in physical activity [69–71]. On the other hand, PE could be considered as a voluntary compensatory behavior. Patients with AN seem to prefer PE as a compensatory behavior rather than purging.

Problematic exercisers had higher MOCI total scores compared to non-problematic exercisers in 2 of the 6 models (significantly for PE-duration and tendency for PE-compulsion; noting that these two dimensions have a huge overlap of problematic exercisers). Davis et al. [49] proposed a model in which obsessive-compulsive personality factors have a direct influence on levels of exercise in AN [20, 46, 49, 51]. They also suggested a positive link between obsessive-compulsive disorders and PE in patients with AN. Davis and Kaptein [46] also found that patients with AN exercising excessively had pronounced obsessive-compulsive disorder symptoms. They used the same duration cut-off (6 hours a week) for their definition of excessive exercise and the same instrument (MOCI) as we did to assess obsessive-compulsive symptomatology. When taking a closer look at the patient’s exercising, it appeared that some patients exercised in a very repetitive and stereotyped manner [72]. Given that exercising for psychological motives (including stress management) is one of the most frequently endorsed motivations for exercise, we could be tempted to hypothesize that this particular aspect of physical activity supports an anxiolytic effect of exercise [73].

Problematic exercisers tended to have lower BSQ scores (reflecting fewer worries about body shape) than non-problematic exercisers when considering PE-intensity. Exercising to control weight and shape is very common in the general population [74] and in eating disorders [56]. It therefore seems that patients found relief for their concerns about body shape when exercising at high intensity levels. Unfortunately, we did not find any previous studies investigating associations between PE (only defined by high intensity) and body image concerns in ED.

Problematic exercisers were found to be significantly older in model 1 (PE-duration) and tended to be younger in model 3 (PE-intensity) than non-problematic exercisers. It seems that older patients preferred exercising for longer periods, while younger patients favored more intensive physical activities. The only study we found that included a large sample of participants (n = 1856) suffering from lifetime ED (including AN, bulimia nervosa and eating disorders not otherwise specified) [26] also found that younger age at study inclusion was associated with PE. In this study, participants were considered problematic exercisers if they reported: 1) that their exercise interfered severely with important activities; 2) exercising for more than 3 hours per day and feeling distressed if unable to exercise; 3) frequently exercising at inappropriate times and places with little or no attempt to overcome this behavior; and 4) exercising despite serious injury, illness or medical complication.

Surprisingly, problematic exercisers had better self-reported quality of life compared to non-problematic exercisers in studies using PE-duration, PE-compulsion and PE-duration+compulsion. Generally, EDs are associated with reduced quality of life compared to healthy controls [75]. To our knowledge, Cook et al. [27] were the only authors to determine the associations between exercise dependence, ED symptomatology and quality of life in healthy individuals [27]. They found that dependence on exercise combined with ED symptoms had a negative impact on quality of life compared to ED symptoms alone. It seems that PE, defined by duration and/or compulsion, could mainly reflect the feeling of a positive impact of exercising on the quality of life reported by our patients.

There were no significant between-group differences in HADS anxiety and depression scores in any of our models. This is in accordance with studies where physical activity was only defined by its characteristics (intensity, duration and/or frequency [16, 54, 76, 77]. This however contrasts with studies that added a notion of compulsion or motivation to exercise in their PE definitions: (1) four studies [16, 18, 23, 25] out of seven [17, 65, 78] found a positive association between PE and depression. (2) Five studies [16–18, 78, 79] out of six [25] found a positive association between PE and anxiety. In fact, previous studies have suggested that PE is a coping strategy to compensate for, remove, and/or alleviate anxiety [17, 18] and depression [18]. Furthermore, it seems that tailored physical activity could reduce anxiety symptoms in the general population [80] and significantly lower scores for depressive symptoms in AN [81]. Further research on this matter is needed.

One of the main strengths of our study was that it identified a large number of definition criteria for PE in the ED literature. Nonetheless, there is a lack of research concerning one criterion that should be taken into consideration when defining PE: dependence on or addiction to exercise. There is growing evidence that exercise should be considered as a dependence or addiction, both in the general population and in animal models. In fact, some authors have hypothesized that changes in the meso-cortico-limbic system [82] and alterations in the dopaminergic system [71] (found in hyperactive animal models mimicking AN) seem to activate brain reward circuits. They could induce addiction to high levels of physical activity. Davis et al. [49] pointed to PE as a type of addictive behavior in AN more than 15 years ago. In spite of this, very little attention has been given to exercise as a potential behavioral addiction in AN. This area has been neglected despite the startling similarities between PE and drug seeking (in substance addiction disorders) or between PE and addictive drugs (on brain reward pathways) [46].

### Limitations

We investigated conjointly psychological and ED-related symptoms linked to exercise in the largest homogeneous clinical sample of inpatients with severe AN studied to date. However, there are three main limitations to take into account: 1) Exercise was assessed using a retrospective semi-structured questionnaire. It is worth noting that this was also the case in more than 75% of the studies in the literature that evaluated physical activity in their AN sample [10]. Since we wanted to investigate associations between PE and a number of explicative factors, we initially intended to recruit a large study sample from 11 treatment units. Using objective measures, such as accelerometers, in such a large cohort would have been extremely hard to monitor and very expensive. Furthermore, we were focusing on inpatients, for whom the use of an objective method to assess physical activity has been associated with problems of refusal or compliance [11]. This is why we chose to use a semi-structured questionnaire to evaluate exercise in our sample. In this case, collecting valid data depended solely on patient recall and accurate report of exercising over a fixed period. The use of this subjective method therefore seemed more appropriate in our sample [83]. 2) The frequency of compulsion to exercise was assessed using a reliable and widely used instrument of ED symptomatology (the EDE-Q). Assessing frequency with a single item (question 18) is a limitation of this study, as mentioned by other authors who performed the same assessment [16]. However, this method has been commonly used in the literature to assess the frequency of hard exercise for weight or shape reasons [24, 25, 56, 58, 59], which is widely known as a compulsion to exercise in the ED literature (S1 Table). 3) The cross-sectional design of our study investigates associations rather than causality.

## Conclusion

A display of abnormally high levels of physical activity has been observed from the earliest clinical descriptions of ED [84]. Since then, researchers have come up with many different terms, definitions and explanatory factors for this phenomenon. As a result, this lack of consistency in terminology and definition has strongly limited all attempts to examine the complex links between physical activity and AN. It has also partly led to the wide prevalence range for PE found in the AN literature. The differing prevalence rates according to the definition of PE implemented should prompt research to determine a valid consensus definition of PE in AN. This could be done, for example, by clearly distinguishing two dimensions of exercise: a quantitative dimension (in terms of frequency, duration and intensity) and a qualitative dimension (the relationship an individual maintains with his/her exercise, and/or its links with ED behaviors, pathological motivation(s) for exercise, compulsion to exercise, exercise dependence or addiction). These two dimensions: 1) do not strictly concern the same patients; 2) probably do not have the same explanatory factors. Concerning instruments: for the quantitative dimension, it is important to use instruments validated in the general population such as the IPAQ [85]. For the qualitative dimension, many instruments previously used to assess compulsion to exercise are linked to ED symptomatology. In future research, it seems necessary to assess different aspects of the qualitative dimension of PE conjointly (obligatory exercise, exercise addiction, commitment to exercise and reasons for exercise) using the appropriate instruments (used in the general population) (i.e. using the obligatory exercise questionnaire, the exercise addiction inventory, the commitment to exercise scale and the reasons for exercise inventory) [16]. Further research on PE as an addiction in AN should be considered [78]. It could be the missing piece of a large jigsaw puzzle entitled “How much exercise is too much in AN?” and help to develop optimal therapeutic approaches.

## Supporting Information

S1 Table Definitions of problematic exercise found in the literature and instruments used for assessment. (DOCX)
