# Supplementary material for: Problematic Exercise in Anorexia Nervosa: Testing Potential Risk Factors against Different Definitions
Source: PLoS One. 2015 Nov 30;10(11):e0143352. doi: 10.1371/journal.pone.0143352 (PMC4664470; doi:10.1371/journal.pone.0143352)
Supplement: S1 Table — (DOCX) [file pone.0143352.s001.docx]

**S1 Table. Definitions of problematic exercise found in the literature and instruments used for assessment.**

| **References** | **Definitions of problematic exercise** | **Instruments used to assess exercise** |
| --- | --- | --- |
| Davis et al. (1995) | Exercise on average, at least 5 hours a week in the year prior to assessment | Interview and physical activity diaries |
| Davis et al. (1997) | The level of physical activity is considerably higher than what is typical for someone of the similar age. If time spent exercising exceeded 1 hour a day at least 6 days a week for a period of no less than 1 month, and if the participant described the exercising as "obsessive," "driven," and "out of control" during the excessive phase. | Interview |
| Davis and Claridge (1998) | -Lifetime exercise status: If time spent exercising exceeded 1 hour a day for at least 6 days a week for a period of no less than 1 month, and if the participant described the exercising as "obsessive," "driven," and "out of control" during the excessive phase.  -Current exercise status: exercise activity at least 6 hours a week averaged over the 12 months prior to assessment. | Interview |
| Davis et al. (1998) | Exercise activity at least 6 hours a week averaged over the 12 months prior to assessment. | Interview |
| Davis et al. (1999) | Exercise activity at least 6 hours a week averaged over 1 month prior to assessment. | Interview |
| Carruth and Skinner. (2000) | Two physical activity levels: ≤ 7 hours/week of physical activity or >7 hours/week of physical activity | Actimeter |
| Solenberger (2001) | “Patients were categorized into high- or low-level exercise groups by a median split of total exercise. The high-level exercise group spent greater than 6.7h/week exercising”. | Clinical charts |
| Penas-Lledo et al. (2002) | Physical exercise at least 5 times a week, for at least 1h without stopping, and with the aim of burning calories. | Clinical charts |
| Davis and Woodside (2002) | Exercise activity for a minimum of 6 hours a week averaged over 1 month prior to assessment. | Interview |
| Davis and Kaptein (2006) | -Lifetime exercise status: If time spent exercising exceeded 1 hour a day for at least 6 days a week for a period of no less than 1 month, and if the participant described the exercising as "obsessive," "driven," and "out of control" during the excessive phase.  -Current exercise status: exercise activity at least 6 hours a week averaged over the 12 months prior to assessment. | Interview |
| Klein et al. (2007) | Exercising at least 6 hours a week, on average. | Accelerometer and interview |
| Dalle Grave et al. (2008) | Compulsive exerciser in case of a positive answer to the first question below and to any of the remaining: “(1) Over the past 4 weeks, have you exercised with the aim of burning up calories to control your shape or weight? (2) Have you felt compelled or obliged to exercise? (3) Have you exercised even when it caused severe interference with important activities? (4) Have you exercised to a level that might be harmful for you? (5) Have you felt distressed if you were unable to exercise?” | Eating disorder examination |
| Mond and Calogero (2009) | “Hard [exercising] as a means of controlling their shape or weight during the preceding 4 weeks”. | Eating disorder examination questionnaire. |
| Bratland-Sanda et al. (2010) | Three criteria: (1) moderate- to-vigorous physical activity if exercising for at least 6 hours a week at admission; (2) persistence of this amount for at least 1 month before admission; (3) classification as exercise dependent symptomatic. | Accelerometer and exercise dependence scale – Revised |
| Bewell-Weiss and Carter (2010) | Minimum of one hour of obligatory exercise aimed at controlling shape and weight, 6 days a week in the month preceding admission. | Eating disorder examination questionnaire |
| Stiles-Shields et al. (2011) | “Over the past four weeks have you exercised as a means of controlling your weight, altering your shape or amount of fat, or burning off calories? Have you felt driven or compelled to exercise?” | Eating disorder examination |
| Smith et al. (2013) | “Hard [exercising] as a means of controlling their shape or weight during the preceding 4 weeks”. | Eating disorder examination questionnaire. |
| Brownstone et al. (2013) | “Hard [exercising] as a means of controlling their shape or weight during the preceding 4 weeks”. | Eating disorder examination questionnaire. |
